# Electronic states and contact ion pair formation in lithium-ion electrolytes investigated by far-ultraviolet spectroscopy and quantum chemical calculations†

**DOI:** 10.1039/d5sc02406d

**Published:** 2025-05-05

**Authors:** Hitomi Sato, Nami Ueno, Ichiro Tanabe

**Affiliations:** a Department of Chemistry, College of Science, Rikkyo University 3-34-1, Nishi-Ikebukuro, Toshima Tokyo 171-8501 Japan itanabe@rikkyo.ac.jp

## Abstract

The electronic states of lithium-ion (Li^+^) electrolytes play a crucial role in understanding their solvation structures and electronic interactions. In this study, far-ultraviolet (FUV) spectroscopy and quantum chemical calculations were used to investigate lithium bis(trifluoromethanesulfonyl)imide (TFSI)-based electrolytes dissolved in dimethyl carbonate (DMC). At low Li^+^ concentrations, the absorption spectra exhibited a redshift, which was attributed to electronic interactions between Li^+^ and DMC in the solvation structure. In contrast, at high concentrations, a clear blueshift was observed. Quantum chemical calculations revealed that this blueshift originates from the formation of contact ion pairs, leading to intermolecular electronic excitation and electron transfer between the TFSI anion and DMC. These findings indicate that the nature of electronic transitions in the FUV region changes significantly with the ionic environment. The results demonstrate that FUV spectroscopy is a powerful technique for probing dynamic changes in solvation and ion association states in electrolytes, offering valuable guidance for the design and optimization of battery materials.

## Abstract 2

Far-UV spectroscopy and TD-DFT calculations probe Li[BF_4_] and Li[TFSI] electrolytes, revealing concentration-dependent spectral shifts: Li[BF_4_] exhibits redshift, while Li[TFSI] transitions from red to blue upon contact ion-pair formation.

## Graphical abstract

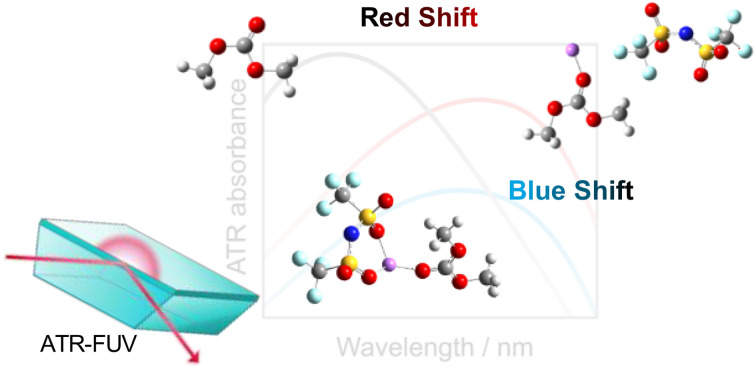

## Introduction

Lithium-ion batteries (LIBs) have rapidly spread as a power source for rechargeable devices such as laptops and mobile phones. The general performance metrics of LIBs, such as power capabilities and lifespans, highly depend on the electrolyte. The ionic conductivity of electrolytes, one of the transport properties that helps to determine the charge and discharge rates of a cell, has been studied.^1–3^ Since the electrolyte plays an important role as a medium for Li^+^ diffusion between the electrodes, it is important to understand the Li^+^ solvation structure.^4^ The solvation structure has been studied using Raman spectroscopy,^5–7^ as well as density functional theory (DFT) and classical molecular dynamics (MD) simulations.^8–10^ Though the most probable coordination number (CN) in the Li^+^ solvation structure is debatable, the CN is most likely 4 from an energetic perspective.^9,10^ In electrolyte solutions, various intermolecular interactions occur, with many Li solvates and free anions present in the low-concentration region, and many contact ion pairs (CIPs) and aggregates (AGGs) formed in the high-concentration region.^11^ In particular, the formation of CIPs is closely related to a change in conductivity at high concentrations.^12,13^ Wang *et al.* reported the formation of CIPs in superconcentrated electrolytes using Raman spectroscopy and DFT-MD simulations.^14^ This study revealed that the formation of CIPs and AGGs at high concentrations retards the diffusion rate of Li^+^.^14^

Dimethyl carbonate (DMC), a representative linear carbonate, is safe to handle due to its low volatility and low toxicity^15^ and has attracted attention as a genuinely green solvent.^16^ Recently, DMC has been used as an electrolyte in LIBs due to its chemical and electrochemical stability, low melting point, high boiling point and high ionic conductivity.^4^ A number of studies have reported on the intermolecular structure, stability, dynamics, and other properties of DMC.^7,17–21^ The Li^+^ solvation structures and CN have also been studied by MD simulations, DFT calculation and Raman spectroscopy.^7,8,10,21^ Lithium bis(trifluoromethanesulfonyl)imide (Li[TFSI]) has attracted much attention because of its potential to improve both chemical and thermal stability as a salt for electrolytes.^22^ Recently, the involvement of [TFSI]^−^ in the solvation structure was reported using Raman spectroscopy and DFT calculations.^10^ In this paper,^10^ it was revealed that Li^+^-(3-DMC)-(1-[TFSI]^−^) is the most possible conformation in concentrated solutions for Li[TFSI]-based electrolytes.

The electronic information of the electrolytes is essentially important because chemical reactions occur in the electronic state. Although ultraviolet (UV) spectroscopy is a powerful method for analyzing the electronic structure without ionizing molecules, there are few reports on this topic. In 2018, Das *et al.* reported the absorption spectra of DMC in the far-ultraviolet (FUV, ≤200 nm) region using synchrotron radiation.^23^ In this report, the spectral bands of DMC were assigned based on quantum chemical calculations. However, it is noteworthy that their spectra were obtained for DMC in the gas phase, which differs significantly from its liquid-state behaviour as an electrolyte. Consequently, direct measurement of FUV absorption spectra for liquid samples and analysis of DMC-Li^+^ interactions remain challenging. In 2007, Higashi *et al.* developed attenuated total reflectance (ATR)-based UV spectroscopy.^24^ In this technique, a deuterium (D_2_) lamp was used as the light source, and the introduction of N_2_ gas was purged along the optical path to exclude the effects of water vapor and oxygen absorption. It allows for the measurement of spectra in the FUV region without requiring a vacuum environment. The light enters the ATR prism, and the evanescent wave generated at the interface between the prism and samples was used as the probe light. By implementing the ATR technology into the FUV region, the spectra in the wavelength range of 145–300 nm can be measured for various materials such as alcohols and alkynes and provide information on various electronic transitions and structures.^25–29^ Furthermore, the combination of spectroscopy and quantum chemical calculations has improved the ability to analyze spectra. Recently, studies on the electronic interactions between Li^+^ and polymers,^30–32^ aqueous solutions^33^ and ionic liquids^34^ using the ATR-FUV method and time-dependent density functional theory (TD-DFT) calculations have been reported. Ueno *et al.* reported the change in the electronic state of water in a highly concentrated aqueous mixed electrolyte solution using ATR-FUV spectra, the derivative method and TD-DFT calculations.^35^ The derivative method is a representative analytical method for separating overlapping peaks. In this report,^35^ second-derivative and third-derivative analyses were applied to the ATR-FUV spectra to separate the overlapping peaks of multiple components and determine the wavelengths of each peak. Additionally, an investigation was conducted on the ionic state of alkali-metal complexes composed of a Li salt and polymers by applying second-derivative analysis to the Raman spectra.^31^

Recently, Sato *et al.* reported the electronic states of DMC and Li^+^DMC using ATR-FUV spectroscopy and TD-DFT calculations.^36^ In this paper,^36^ DMC involved in the Li solvation structure was defined as Li^+^DMC and DMC not involved was defined as free DMC, respectively. In the ATR-FUV spectra of the Li-DMC electrolytes, a decrease in the absorption intensity of free DMC (∼150 nm) and a corresponding increase in that of Li^+^DMC (∼155 nm) were observed with increasing Li^+^ concentration. The area ratio of the ATR spectral intensities corresponding to free DMC and Li^+^DMC indicated a uniform solvation structure in the low-concentration region (≤1.5 M in this study). Furthermore, the redshift was attributed to the difference in wavelengths between the intramolecular excitation of DMC and the electronic transition from DMC to Li^+^. This study represents the first investigation of the electronic state of liquid-phase DMC in the FUV region, which is inaccessible by conventional electronic absorption spectroscopy.

In this study, lithium tetrafluoroborate (Li[BF_4_]) and Li[TFSI] solutions over a wide concentration range (*i.e.*, 0.0–6.0 M) were investigated to clarify the Li^+^ concentration-dependent solvation structures and their corresponding electronic states. Absorption in the FUV region associated with electronic excitation and electron transition in the concentrated Li^+^ electrolytes was revealed by ATR-FUV spectroscopy combined with TD-DFT calculations. In the case of Li[BF_4_] solutions, a redshift was observed across the entire concentration range in the ATR-FUV spectra. In contrast, Li[TFSI] solutions exhibited a change from redshift to blueshift at high concentrations. TD-DFT calculations indicated that the blueshift in the concentrated Li[TFSI] solutions was attributed to the formation of CIPs. The detection of FUV absorption and the analysis of CIP electronic states, which involve both intramolecular and intermolecular electronic excitation, cannot be achieved using conventional electronic absorption spectroscopy. The insights gained from this study on electronic states are expected to contribute to elucidating the mechanisms of electrode reactions in LIBs, such as the formation of the solid electrolyte interphase (SEI), and to advancing the performance of lithium-ion batteries.

## Experimental

### ATR-FUV measurements

**Fig. 1:**
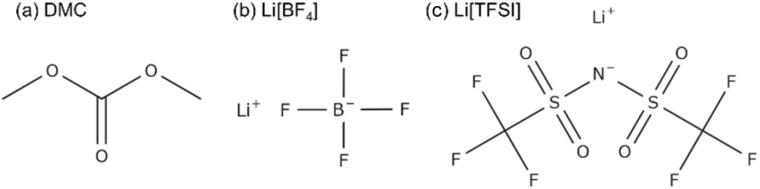
Chemical structures of reagents. Chemical structures of (a) dimethyl carbonate (DMC), (b) Li[BF_4_], and (c) Li[TFSI].

The ATR-FUV absorption spectra were recorded in the 145–300 nm wavelength range at 0.1 nm intervals (scan rate = 150 nm min^−1^ and incidence angle = 70°). A sapphire prism (optical path length = 8 mm, UV grade, Optoline, Tokyo, Japan) was used as the internal reflection element (IRE). The reagents (DMC, Li[BF_4_] and Li[TFSI]; their chemical structures are shown in Fig. 1) were purchased from Kanto Chemical Co., Inc. (Tokyo, Japan) and used without further purification. Li[BF_4_] and Li[TFSI] solutions with 13 different Li^+^ concentrations (0.0, 0.5, 1.0, 1.5, 2.0, 2.5, 3.0, 3.5, 4.0, 4.5, 5.0, 5.5 and 6.0 M) were prepared. These solutions were placed on the sapphire prism, and ATR absorption was defined as −log(*I*/*I*_0_), where *I* and *I*_0_ represent the reflected light intensity in the presence and absence of the sample on the ATR prism, respectively.

### TD-DFT calculations

Spectral assignments were performed using DFT and TD-DFT calculations. In this study, 11 models (*i.e.*, free DMC, Li^+^DMC, Li solvates (CN = 2–4), free [BF_4_]^−^, Li[BF_4_], free [TFSI]^−^, Li[TFSI], and two types of CIP ([BF_4_]^−^Li^+^DMC and [TFSI]^−^Li^+^DMC)) were investigated. Energy minimization of the ground states was performed using the B3LYP/aug-cc-pVTZ basis set. Their vertical transition energies and oscillator strengths were calculated using the TD-CAM-B3LYP/aug-cc-pVTZ basis set. All calculations were performed using the Gaussian16 program. To calculate the molar extinction coefficient (*ε*), the oscillator strength was multiplied by the energy width of 0.5 eV for each transition. All molecular orbitals were obtained using the GaussView graphical interface with an equivalent number set of 0.02.

## Results and discussion

### ATR-FUV spectra of Li[BF_4_]-DMC solutions

**Fig. 2:**
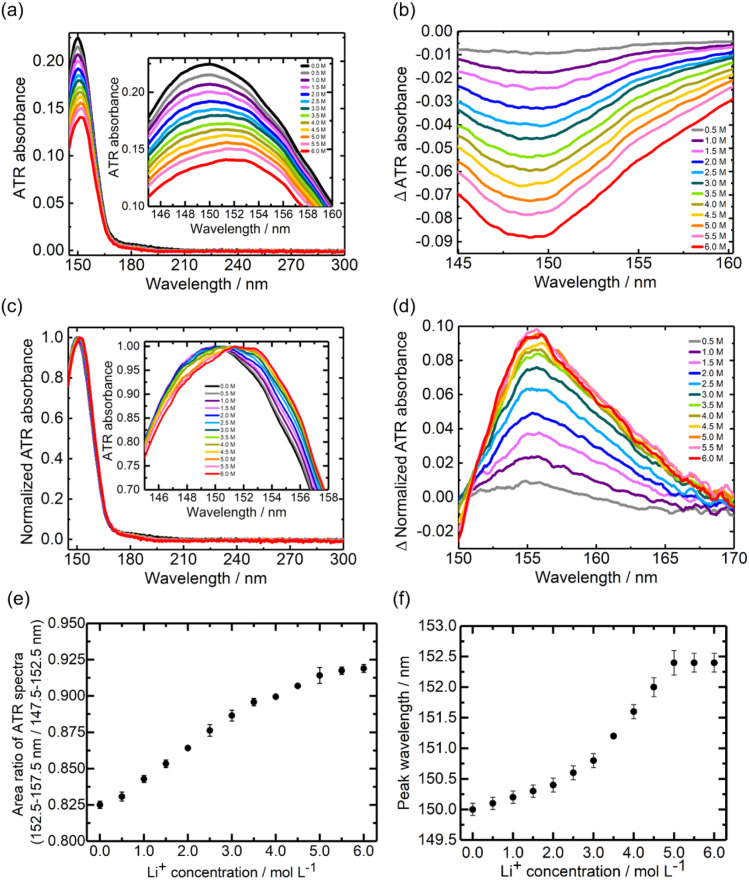
ATR-FUV spectra and analysis of Li[BF_4_]. (a) ATR-FUV absorption spectra of pure DMC (black line) and Li[BF_4_] DMC solutions (colored lines) and (b) their difference spectra in the 145–300 nm spectral region. (c) Normalized spectra of (a) and (d) their difference spectra. (e) Li^+^ concentration dependence of the area ratio of the ATR spectral intensity in 152.5–157.5 nm to 147.5–152.5 nm, and (f) the peak wavelength *vs.* the Li^+^ concentration in (a). The standard deviation bar was calculated from the results of three ATR measurements.

Fig. 2a and b show the ATR-FUV spectra and their difference spectra of Li[BF_4_] in DMC solvent within the 145–300 nm range, respectively. As reported previously for the low Li[BF_4_] concentration (0.0–1.5 M) case,^36^ an absorption peak associated with free DMC was detected around 150 nm, and the intensity decreases with increasing Li^+^ concentration. It should be noted that the acquired ATR-FUV spectra were used without the Kramers–Kronig transform (KKT), which separates the effects of the extinction coefficient (*κ*) and the refractive index (*n*). The ATR spectrum includes the effect of *κ* and *n* in the sample and the sapphire prism.^24^ As a result, the ATR spectra are different from the transmission spectrum, which considers only the effect of *κ*. Recently, studies applying the KKT to ATR-FUV spectra were reported, and correct features of the absorption spectra in the FUV region were confirmed.^37^ However, in this study, the absorbance of the DMC is strong and spectral intensity is not zero at the edge of the measurement wavelength range. Moreover, the complex spectral changes dependent on Li^+^ concentration introduced significant challenges for the application of KKT. Consequently, in the following discussion, the acquired ATR-FUV spectra were used without KKT.

### Quantitative relationship between free DMC and Li^+^DMC in Li[BF_4_]-DMC solutions

Fig. 2c and d show the normalized ATR-FUV spectra (Fig. 2a) and their difference spectra, respectively. Fig. 2d reveals an increase in the absorption band around 155 nm with the addition of Li^+^, which was assigned to the absorption of Li^+^DMC.^36^ As discussed in a previous study,^36^ by calculating the absorption intensity ratio between 150 nm and 155 nm, the relative amounts of free DMC and solvated DMC (Li^+^DMC) can be analyzed. As discussed in Fig. S1,† the peak wavelength was also estimated from the second-derivative spectra in this study. Fig. 2e shows the Li^+^ concentration dependence of the area ratio of the ATR spectral intensity between 152.5–157.5 nm and 147.5–152.5 nm in Fig. 2a. Up to a concentration of 3.5 M, the area ratio was linearly related to the Li^+^ concentration, indicating that the abundant free DMC coordinates to form Li^+^DMC upon the addition of Li^+^. When the Li^+^ concentration exceeds 3.5 M, the slope decreases, and at concentrations above 5.0 M, it remains almost constant.

These results indicate that the amount of change in the ratio of free DMC to Li^+^DMC becomes smaller with the addition of Li^+^, which is considered to be due to the decrease in the quantity of free DMC in the solution. The CN of Li^+^ has been reported to be ∼4 in Raman scattering measurements and MD simulations.^7,8,10,21^ In this experiment, at a Li[BF_4_] concentration of 4.0 M, the molar ratio of Li^+^ to DMC is estimated to be approximately 4. That is, the change in the ratio of free DMC to Li^+^DMC with respect to Li^+^ concentration around 4.0 M is reasonable. In TD-DFT calculations, for all coordination numbers (CN = 1–4) of DMC to Li^+^, the absorption wavelength was calculated to be longer than that of free DMC (Fig. S2†).

### Peak wavelength shifts in Li[BF_4_]-DMC solutions

As noted above, the ATR-FUV spectra showed a redshift with Li^+^ addition. Fig. 2f depicts the peak position of the ATR-FUV spectra (Fig. 2a) *vs.* the Li^+^ concentration. As the Li^+^ concentration increases, the peak position shifts to longer wavelengths and remains almost constant at concentrations above 5.0 M. The behaviour of the peak wavelength shifts with respect to Li^+^ concentration is consistent with the change in the ratio of the absorption intensities originating from free DMC and Li^+^DMC (Fig. 2e). The area ratio (Fig. 2e) and derivative-based peak wavelength (Fig. 2f) are distinct metrics: the former integrates band intensities over fixed ranges, whereas the latter identifies inflection points, leading to small deviations between them. The effects of [BF_4_]^−^, Li[BF_4_], and the CIP ([BF_4_]^−^Li^+^DMC) are shown in Fig. S3,† where it was concluded that they have negligible impact on the spectral changes of the Li[BF_4_]-DMC solutions.

### ATR-FUV spectra of Li[TFSI]-DMC solutions

**Fig. 3:**
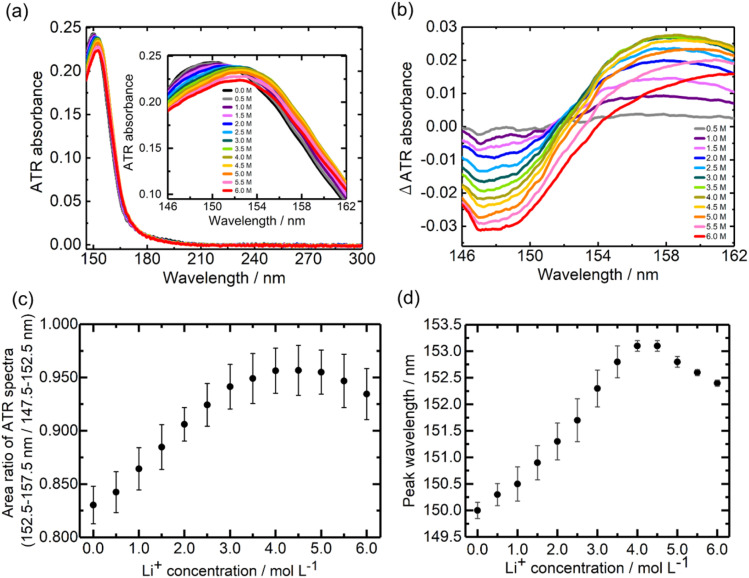
ATR-FUV spectra and analysis of Li[TFSI]. (a) ATR-FUV absorption spectra of pure DMC (black line) and Li[TFSI] DMC solutions (colored lines) and (b) their difference spectra in the 145–300 nm spectral region. (c) Li^+^ concentration dependence of the area ratio of the ATR spectral intensity between 152.5–157.5 nm and 147.5–152.5 nm, and (d) the peak wavelength *vs.* the Li^+^ concentration in (a). The standard deviation bar was calculated from the results of three ATR measurements.

Fig. 3a and b show the ATR-FUV spectra and their difference spectra of Li[TFSI] in DMC solvent in the 145–300 nm range. The difference spectra (Fig. 3b) exhibit a decrease in absorption around 148 nm with the addition of Li^+^. Additionally, the spectral intensity around 158 nm increased in the range of 0.0–4.0 M, while it decreased in the 4.0–6.0 M range with increasing Li^+^ concentration. The normalized spectra (Fig. S4a†) showed a redshift up to 4.0 M, followed by a blueshift upon further addition of Li^+^ above 4.0 M. The difference spectra (Fig. S4b†) indicate an increase in spectral intensity around 158 nm in the range of 0.0–4.0 M, with a subsequent decrease in the intensity after 4.0 M. Similar to the case of Li[BF_4_]-DMC solutions, the Li^+^ concentration dependence of the area ratio between 152.5–157.5 nm (Li^+^DMC) and 147.5–152.5 nm (free DMC) in the ATR-FUV spectra was calculated. As shown in Fig. 3c, the area ratio increased up to a Li^+^ concentration of 5.0 M and decreased at higher concentrations.

Fig. 3d presents the relationship between the peak wavelength of the ATR-FUV spectra (Fig. 3a) and the Li^+^ concentration. In the range of 0.0–4.0 M, the peak position shifted to longer wavelengths with increasing Li^+^ concentration, whereas beyond 4.0 M, it shifted to shorter wavelengths. These spectral behaviours in Fig. 3 differ significantly from that observed in Li[BF_4_]-DMC solutions, which was discussed in the previous section. In particular, the short-wavelength shift observed in the high-concentration region in Li[TFSI] solutions, which was not seen in Li[BF_4_] solutions, is an important finding of this study.

### Effects of absorption of [TFSI]^−^

**Fig. 4:**
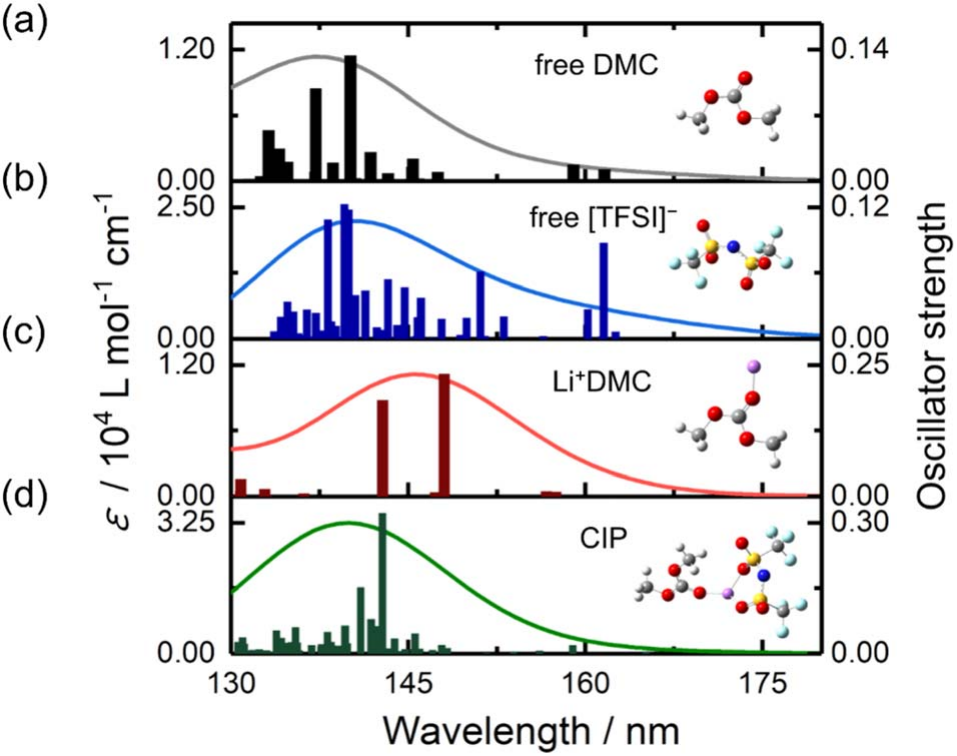
Theoretical analysis of spectral changes. Time-dependent density functional theory-calculated oscillation strengths and molar extinction coefficients (*ε*) of (a) free DMC, (b) free [TFSI]^−^, (c) Li^+^DMC and (d) the CIP ([TFSI]^−^Li^+^DMC).

A comparison of Fig. 2a (Li[BF_4_]-DMC solutions) and Fig. 3a (Li[TFSI]-DMC solutions) reveals that the decrease in absorption around 150 nm is significantly smaller in Li[TFSI] solutions. To elucidate the cause of this phenomenon, TD-DFT calculations were conducted (Fig. 4). The results indicate that free [TFSI]^−^ (Fig. 4b) exhibits absorption around 150 nm, where free DMC (Fig. 4a) also absorbs. As a result, the reduction in absorption intensity around 150 nm was suppressed upon the addition of Li[TFSI]. Furthermore, at wavelengths longer than the absorption peak of free DMC, it was suggested that the absorption is contributed not only by Li^+^DMC (Fig. 4c) but also by free [TFSI]^−^ (Fig. 4b). From these results, it was found that, in Fig. 3c, both wavelength regions (*i.e.*, 147.5–152.5 nm and 152.5–157.5 nm) contain the absorption of free [TFSI]^−^. Therefore, the positive relationship between the area ratio and Li⁺ concentration up to 4.0 M (Fig. 3c) indicates the decrease of free DMC and the increase of Li^+^DMC and free [TFSI]^−^.

### Effects of the CIP in high concentration Li[TFSI]-DMC solutions

As mentioned above, in high-concentration Li[TFSI] solutions above 4.0 M, the peak wavelength shifted to shorter wavelengths. This phenomenon cannot be explained solely by considering free DMC, Li^+^DMC, and free [TFSI]^−^. While the potential impact of Li[TFSI] was discussed in Fig. S5,† the following discussion will focus on the CIP ([TFSI]^−^Li^+^DMC).

To investigate the contribution of the CIP ([TFSI]^−^Li^+^DMC), TD-DFT calculations were performed (Fig. 4d). Interestingly, the absorption peak wavelength of the CIP was calculated to be shorter than that of Li^+^DMC. Additionally, compared to free [TFSI]^−^, the absorption on the longer wavelength side was not significant. The formation of the CIP ([TFSI]^−^Li^+^DMC) reduces the proportion of Li^+^DMC and free [TFSI]^−^ that contribute to longer-wavelength absorption. That is, the peak wavelength of the solution shifts to shorter wavelengths, as shown in Fig. 3a and d. In addition, the decrease in the intensity ratio between 152.5–157.5 nm and 147.5–152.5 nm at high concentrations shown in Fig. 3c can also be explained. Here, it should be noted that the TD-DFT calculations employ isolated cluster models (*e.g.*, free DMC, free [TFSI]^−^, Li^+^DMC, and CIP) and therefore do not directly reproduce the experimental 0.0–6.0 M solution. Nevertheless, the computational results qualitatively reproduce the experimental observations well, indicating that the local coordination environment is the dominant factor governing the spectral changes. In Fig. S6,† the peak wavelength of the CIP was estimated from the second-derivative spectra. As a result, an absorption peak attributed to the CIP was found at 153.6 nm, which lies between the wavelengths of free DMC (∼150 nm) and Li^+^DMC (∼155 nm). As described in the Introduction, it is well known that a CIP is formed in high-concentration solutions,^11–14^ and thus these results and interpretations are reasonable. According to the MD simulations,^34^ solvent molecules and ions coordinating with Li^+^ exchange on a sub-nanosecond timescale. By contrast, the FUV spectra reported here reflect the time-averaged solvation structure over seconds. Our spectral analysis demonstrates that the fraction of the CIP in solution increases at high concentrations. In other words, at low Li^+^ concentrations, the number of counter anions ([TFSI]^−^) is small relative to solvent molecules (DMC), so CIP formation is not favored; however, as the concentration increases, CIP formation becomes more likely. In this study, the electronic excitations are induced by the incident light used in the ATR-FUV spectral measurements, and the electronic states within the CIP are elucidated through the spectra and TD-DFT calculations. Previous Raman spectroscopy studies have provided valuable insights into CIP formation *via* vibrational markers.^13,14^ However, Raman probes vibrational motions between atoms in a molecule, whereas our ATR-FUV approach directly monitors electronic excitations associated with the CIP. The complementary nature of these techniques can offer a more complete picture of solvation and ion-pairing phenomena.

### Electronic states of the CIP

**Fig. 5:**
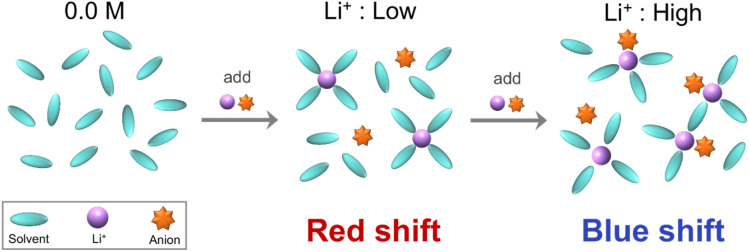
Proposed ion behaviours. Schematic diagram of the peak wavelength shifts and the corresponding ion behaviour in Li[TFSI] solution.

**Fig. 6:**
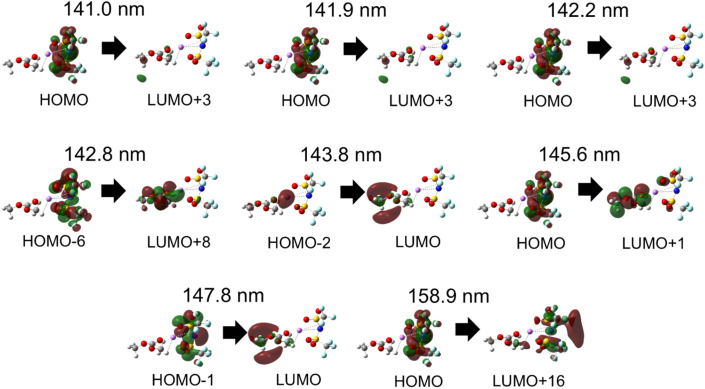
Simulated electronic states in the CIP ([TFSI]^−^Li^+^DMC). Main initial and final molecular orbitals of the electronic transitions with oscillator strengths above 140 nm from the TD-DFT calculations of the CIP (Fig. 4d).

The peak wavelength shifts in Li[TFSI] solutions and the corresponding ion behaviour, as demonstrated in the previous sections, are summarized in Fig. 5. First, when Li[TFSI] is added to pure DMC, the amount of free DMC decreases, and the peak wavelength shifts to longer wavelengths due to the contributions of Li^+^DMC and free [TFSI]^−^. As the concentration increases, a CIP is formed, which absorbs at shorter wavelengths than Li^+^DMC and free [TFSI]^−^, leading to a blueshift in the peak wavelength. The detection of the formation of the CIP through FUV spectra, which arises from the electronic excitation and electron transfer of molecules and ions, is a key aspect of this study. Fig. 6 presents the initial and final molecular orbitals corresponding to the eight largest oscillator strengths above 140 nm from the TD-DFT calculations of the CIP shown in Fig. 4d.

The largest calculated electronic transition at 142.8 nm is mainly due to intermolecular excitation from [TFSI]^−^ to DMC. This can be interpreted as an electronic interaction between [TFSI]^−^ and DMC mediated by Li^+^. Additionally, as seen in the electronic transition at 143.8 nm, there is also absorption associated with intermolecular electron transfer from Li^+^ to DMC. On the other hand, the longest-wavelength electronic transition primarily originates from intramolecular electronic excitation within [TFSI]^−^. More details of the TD-DFT results are summarized in Tables S1–S3 and Fig. S7–S9.† Consequently, in Li[TFSI] solutions, both intramolecular and intermolecular absorptions are present. Studying the electronic states of molecules in Li^+^ electrolytes may contribute to elucidating the reaction mechanisms involved in SEI formation. Currently, research is underway on the changes in ATR-FUV spectra of Li[TFSI] and Li[BF_4_] under voltage application to electrodes.

## Conclusions

In this work, we revealed the Li^+^ concentration dependence of the ratio of the ATR spectral intensity ratio between 152.5–157.5 nm (Li^+^DMC) and 147.5–152.5 nm (free DMC) for 0.0–6.0 M Li[BF_4_]-DMC solutions. In the Li^+^ concentration range above 5.0 M, this ratio remains constant, and the redshift of the peak wavelength correspondingly stops in the high-concentration region. On the other hand, the ATR spectral intensity ratio in 152.5–157.5 nm to 147.5–152.5 nm, which increases at low concentrations, decreases in high-concentration Li[TFSI] solutions. The peak wavelength, which redshifted up to 5.0 M, began to blueshift at higher concentrations. The TD-DFT calculation results suggested that this was due to the formation of a CIP at high concentrations. This study successfully detected the formation of CIP resulting from the electronic excitation and electron transfer of molecules and ions using ATR-FUV spectroscopy. It was also revealed that the main electronic transition of the CIP was due to intermolecular excitation from [TFSI]^−^ to DMC. This study clarified the absorption in the FUV region associated with electronic excitation and electronic transition in Li^+^ electrolyte using ATR-FUV spectroscopy and TD-DFT calculations. Understanding the electronic states of electrolytes under various ionic conditions provides a fundamental basis for unravelling complex electrochemical processes, including charge transfer dynamics and interphase formation at electrode surfaces. Understanding how CIP formation modulates FUV electronic transitions provides a new way to correlate solvation structures with ionic conductivity and SEI composition, thereby guiding the rational design of more efficient and durable lithium-ion battery electrolytes. These insights pave the way for designing more efficient and durable lithium-ion battery systems by tailoring electrolyte compositions to control interfacial reactions and long-term stability. As a next step, *in situ* ATR-FUV measurements under applied potential will be pursued to directly observe electronic state changes during charge–discharge cycles, with potential applications in polymer-gel and solid-state electrolytes.

## Author contributions

H. S. mainly performed spectroscopic experiments, simulations, and data analysis. N. U. conducted the data analysis. I. T. conducted the present study. All authors discussed the results and contributed to the manuscript preparation.

## Conflicts of interest

There are no conflicts to declare.

## Supplementary Material

SC-016-D5SC02406D-s001

## Data Availability

The data supporting the findings of this study are available within the article or ESI.†
