# Supplementary material for: Electronic states and contact ion pair formation in lithium-ion electrolytes investigated by far-ultraviolet spectroscopy and quantum chemical calculations
Source: Chem Sci. 2025 May 5;16(25):11232–9. doi: 10.1039/d5sc02406d (PMC12076015; doi:10.1039/d5sc02406d)
Supplement: SC-016-D5SC02406D-s001 [file SC-016-D5SC02406D-s001.pdf]

Supplementary Information (SI) for Chemical Science.
This journal is © The Royal Society of Chemistry 2025

Electronic states and contact ion pair formations in lithium-ion electrolytes investigated by far-ultraviolet spectroscopy and quantum chemical calculations

Hitomi Sato, Nami Ueno* and Ichiro Tanabe*

SI 1. Second-derivative analysis of Li[BF_4]-DMC solutions.
SI 2. TD-DFT calculations for DMC and solvated DMC with *CN* = 1-4.
SI 3. Effects of free [BF_4]$^-$, Li[BF_4] and ([BF_4]$^-$$Li^+$DMC) on ATR-FUV spectra.
SI 4. Normalized ATR spectra for Li[TFSI] solution.
SI 5. Effect of Li[TFSI] on ATR spectra.
SI 6. Second-derivative analysis of Li[TFSI]-DMC solutions.
SI 7. Details of TD-DFT calculations for DMC, Li^+DMC and CIP ([TFSI]$^-$$Li^+$DMC).

SI 1. Second-derivative analysis of $Li[BF_4]$-DMC solutions.

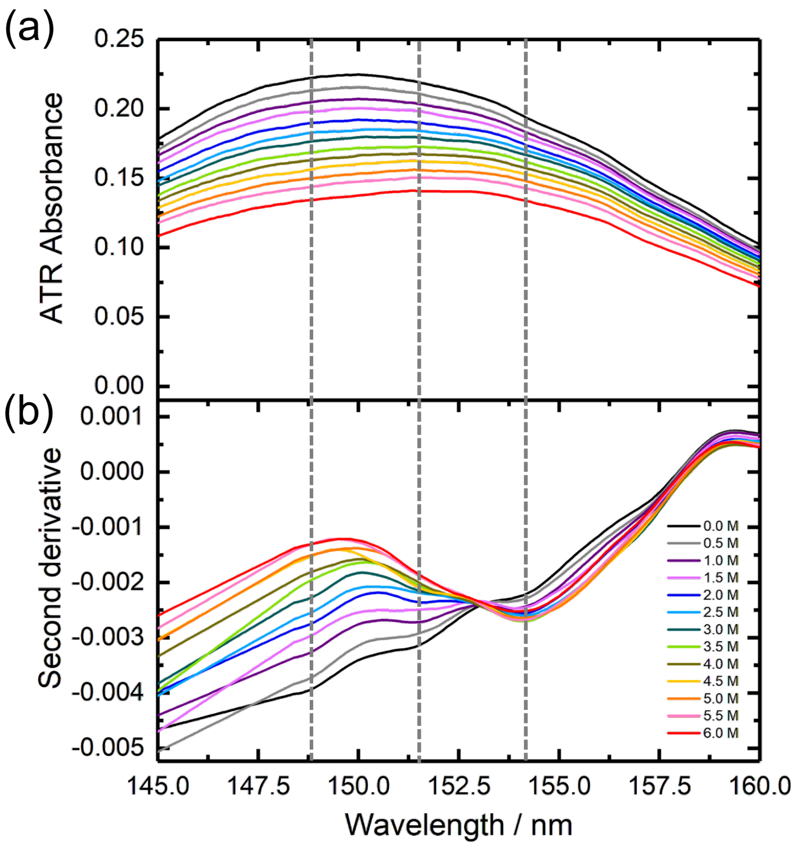

Fig. S1 (a) ATR-FUV and **(b)** their second-derivative spectra of $Li[BF_4]$-DMC solutions. The vertical broken lines indicate the peak wavelength in the second-derivative spectra at 148.8, 151.4, and 154.2 nm.

The derivative method was used to separate and analyze the overlapping peaks. The derivative method is a generally used analytical method to detect the peak wavelength of the broadband with some overlapped bands. The local minimum of the second-derivative spectra show the original curve's inflection point and peak wavelength of the absorption. In this study, the peak wavelengths of the ATR-FUV spectra were determined by the local minimum of the second-derivative spectra using the Savitzky–Golay (SG) method with 71 window points. Fig. S1a and b show the ATR-FUV and their second-derivative spectra, respectively. It should be noted that the peak top of the second-derivative spectra is negative. As shown in Fig. S1b, pure DMC (0.0 M, black line) exhibits two peaks at approximately 148.8 nm and 151.4 nm, which correspond to a spectrum with a peak at around 150 nm in the original spectrum. By adding Li^+, these two peaks in the second-derivative spectra decrease in intensity, and the peak at 154.2 nm becomes more prominent, which is attributed to Li^+DMC. Therefore, in this study, the integrated ATR spectral intensities in the 152.5–157.5 nm and 147.5–152.5 nm ranges were used to estimate the absorption due to free DMC and Li^+DMC, respectively.

SI 2. TD-DFT calculations for DMC and solvated DMC with *CN* = 1-4.

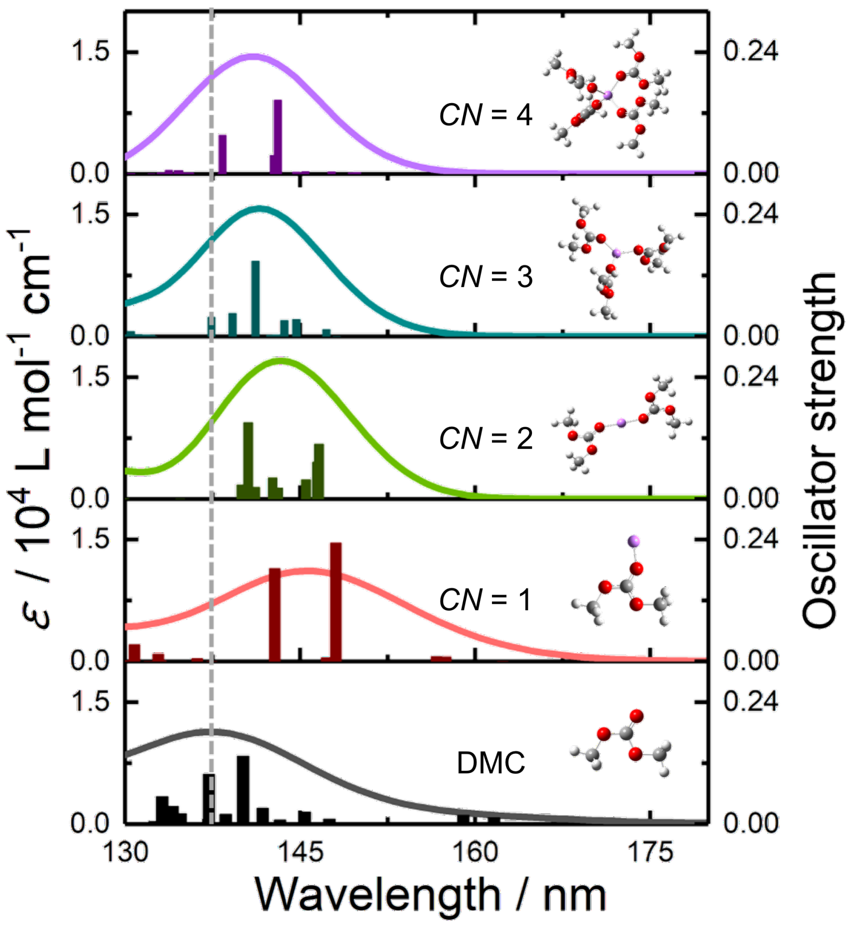

Fig. S2 Time-dependent density functional theory (TD-DFT) calculated oscillator strengths and molar extinction coefficients (ε) of DMC and solvated DMC with coordination number (*CN*) = 1-4. This TD-DFT calculation was performed using the TD-CAM-B3LYP/aug-cc-pVTZ basis set. Based on the calculated vertical transition energies and oscillator intensities, the absorbance spectra were simulated assuming an energy width of 0.5 eV for each transition. The oscillator strength and molar extinction coefficient (ε) of the solvated DMC are divided by the coordination number of DMC. Vertical broken lines indicate peak wavelengths of the simulated spectrum of DMC.

Our research group recently reported the TD-DFT calculation results for free DMC and Li^+DMC,[S1] revealing that Li^+DMC exhibited absorption at longer wavelengths compared to free DMC. This result is consistent with the ATR-FUV spectral measurements. However, previous studies only considered the calculation results for a coordination number (*CN*) = 1, corresponding to one DMC and one Li^+ in the simulation model. It is known that in electrolytes, Li^+ is typically solvated by four solvent molecules. Therefore, in this study, TD-DFT calculations were performed for models with *CN* = 1-4. As when in Fig. S2, TD-DFT calculations revealed that all Li solvated DMC exhibited absorption at longer wavelengths than free DMC. Consequently, regardless of the coordination number of the Li solvates, the results of the ATR measurements are supported.

SI 3. Effects of free $[BF_4]^-$, $Li[BF_4]$ and CIP ($[BF_4]^-Li^+DMC$) on ATR-FUV spectra.

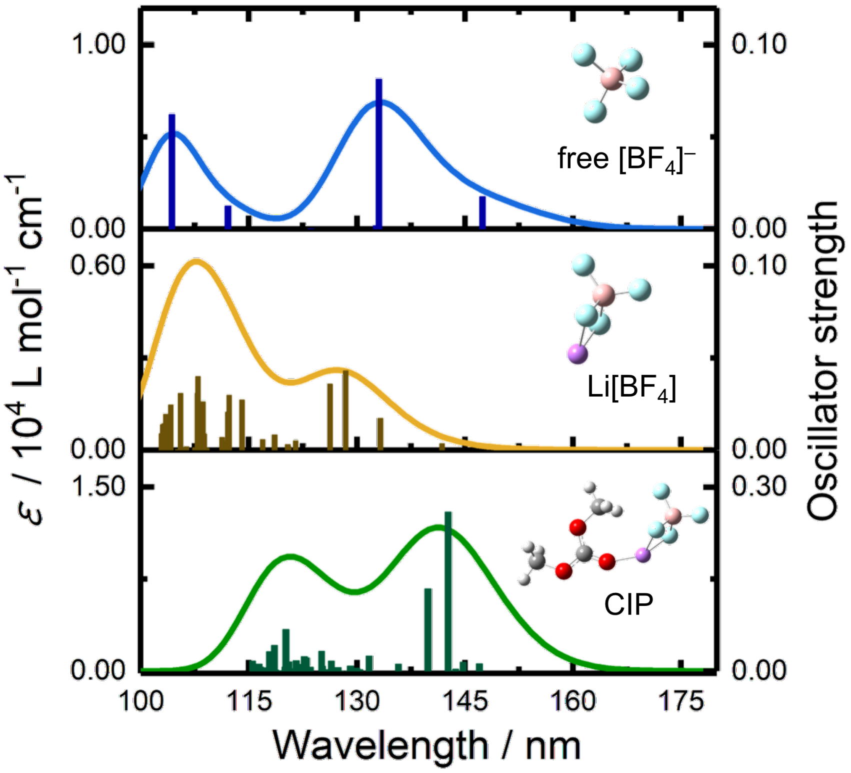

Fig. S3 Time-dependent density functional theory (TD-DFT) calculated oscillator strengths and molar extinction coefficients (ε) of free $[BF_4]^-$, $Li[BF_4]$, and CIP ($[BF_4]^-Li^+DMC$). TD-DFT calculations were performed using the TD-CAM-B3LYP/aug-cc-pVTZ basis set. Based on the calculated vertical transition energies and oscillator intensities, the absorbance spectra were simulated assuming an energy width of 0.5 eV for each transition.

Fig. S3 shows the calculated oscillator strengths and molar extinction coefficients (ε) of free $[BF_4]^-$, $Li[BF_4]$, and CIP ($[BF_4]^-Li^+DMC$). These three species exhibit absorptions below 160 nm. Compared to DMC and solvated DMC shown in Fig. S2, ε of free $[BF_4]^-$ and $Li[BF_4]$ are relatively low. In contrast, ε of CIP ($[BF_4]^-Li^+DMC$) is close to those of DMC and solvated DMC. However, the influence of CIP ($[BF_4]^-Li^+DMC$) did not be detected in the ATR-FUV spectra, which may be due to its low formation or the difficulty in distinguishing its absorption from that of free DMC due to their close wavelengths. While the exact cause is not clear, the influence of CIP ($[BF_4]^-Li^+DMC$) was not considered in this study. Notably, as discussed later, in the case of Li[TFSI], both free $[TFSI]^-$ and the formation of CIP ($[TFSI]^-Li^+DMC$) had a significant impact on the ATR-FUV spectra, which is a key aspect of this study.

SI 4. Normalized ATR spectra for Li[TFSI] solution.

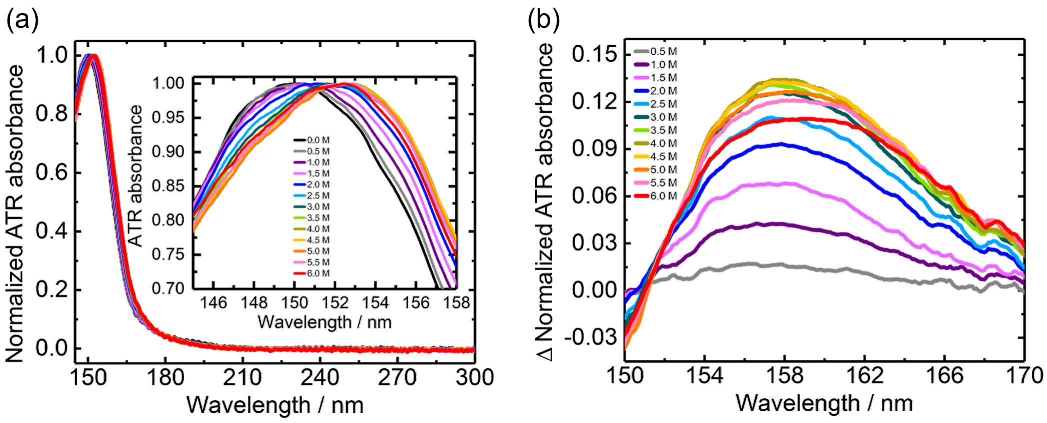

Fig. S4 (a) Normalized spectra of pure DMC (black line) and Li[TFSI] DMC solutions (colored lines) and (b) their difference spectra.

The ATR spectra in Fig. 3a were normalized to the maximum peak intensity (Fig. S4a), showing that the peak wavelength redshift with increasing Li^+ concentration up to 4.0 M. Additionally, after 4.0 M, the peak wavelength slightly blueshift with increasing Li^+ concentration. In their difference spectra (Fig. S4b), the absorption on the longer wavelength side increased up to 4.0 M, and then decreased after 4.0 M. This reversal of the trend was not observed in the case of Li[BF_4]. Identifying the cause of this change is a key subject of the present study.

SI 5. Effect of Li[TFSI] on ATR spectra.

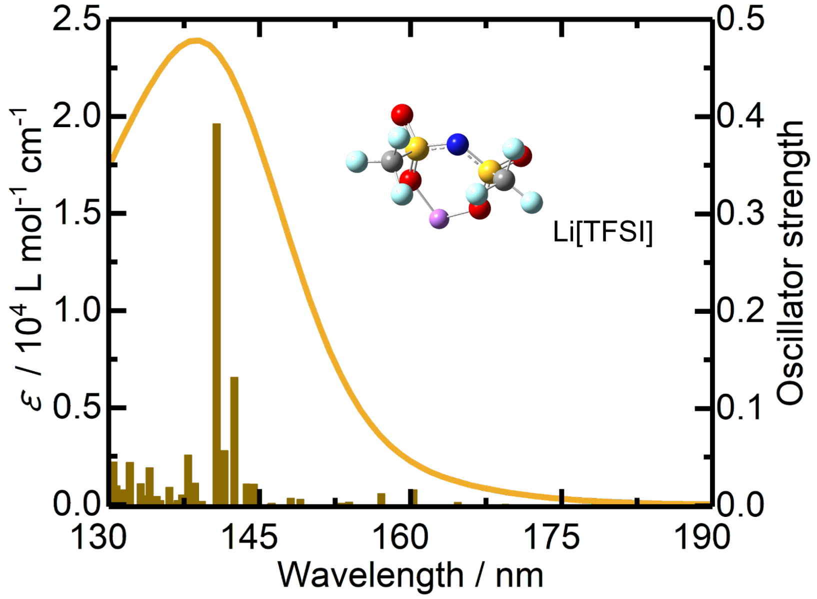

Fig. S5 Time-dependent density functional theory (TD-DFT) calculated oscillator strengths and molar extinction coefficients (ε) of Li[TFSI]. This TD-DFT calculation was performed using the TD-CAM-B3LYP/aug-cc-pVTZ basis set. Based on the calculated vertical transition energies and oscillator intensities, the absorbance spectra were simulated assuming an energy width of 0.5 eV for each transition.

Fig. S5 shows the calculated oscillator strengths and molar extinction coefficients (ε) for Li[TFSI]. Li[TFSI] exhibits absorption in a wavelength range similar to those of free DMC (Fig. 4a) and CIP ([TFSI]$^-$Li$^+$DMC) (Fig. 4d). However, in the solution, Li[TFSI] is solvated by DMC, leading to the formation of CIP ([TFSI]$^-$Li$^+$DMC). Therefore, in the discussion regarding the electrolyte, the focus is placed not on only Li[TFSI] model but on CIP ([TFSI]$^-$Li$^+$DMC) model.

SI 6. Second-derivative analysis of Li[TFSI]-DMC solutions.

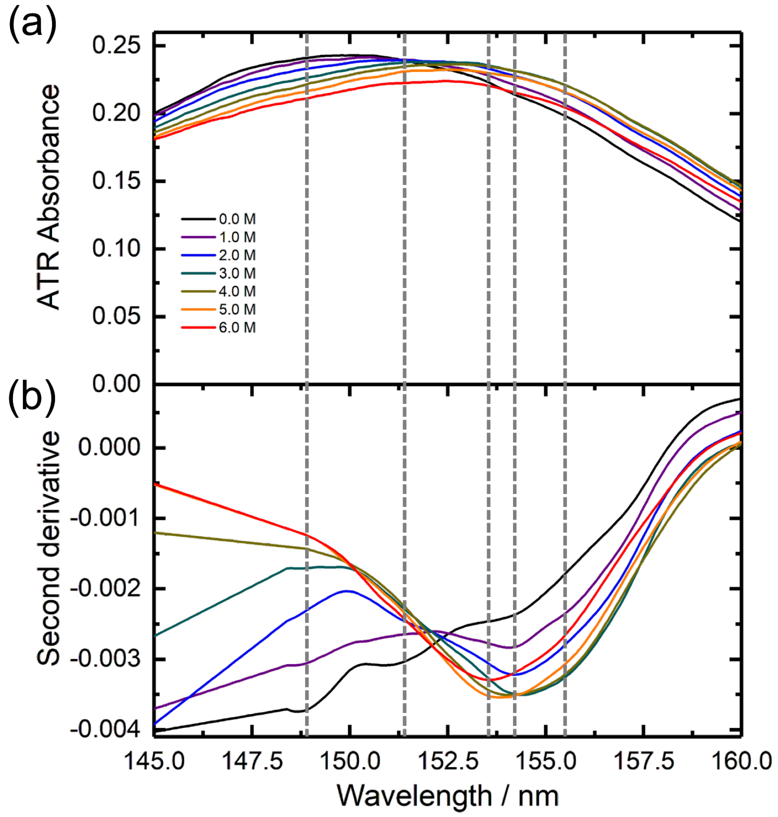

Fig. S6 (a) ATR-FUV and (b) their second-derivative spectra of Li[TFSI]-DMC solutions. The spectra were displayed at 1.0 M intervals for better clarity. The vertical broken lines indicate the peak wavelength of in the second-derivative spectra at 148.8, 151.4, 153.6, 154.2, and 155.5 nm.

Fig. S6a and b show the ATR-FUV and their second-derivative spectra. It should be noted that the peak top of the second-derivative spectra is a negative value. As described in SI 1, in the second-derivative spectra (Fig. S1b), pure DMC (0.0 M, black line) exhibits two peaks at approximately 148.8 nm and 151.4 nm, which are attributed to free DMC, and the peak at 154.2 nm is assigned to Li^+DMC.

Additionally, two other peaks were detected at 153.6 and 155.5 nm, despite being somewhat difficult to distinguish clearly. The peak at 155.5 nm gradually became larger and more distinct with the addition of Li^+, and slightly decreased at higher concentrations of above 5.0 M, similar to the 154.2 nm peak attributed to Li^+DMC. Therefore, it was concluded that this peak originates from the absorption of free $[TFSI]^-$. According to the TD-DFT calculation results shown in Fig. 4b, in addition to the main absorption peak on the shorter wavelength side, a relatively strong electronic transition with a high oscillator strength was observed on the longer wavelength side, which may be detected by the FUV measurements.

On the other hand, the 153.6 nm peak was barely observed at low concentrations and became prominent at concentrations of 5.0 M or more. Therefore, it was concluded that this absorption is due to CIP ($[TFSI]^-Li^+$DMC). This wavelength is located on the shorter wavelength side of the Li^+DMC absorption, leading to the observed short-wavelength shift in the ATR-FUV spectrum upon the formation of CIP ($[TFSI]^-Li^+$DMC).

SI 7. Details of TD-DFT calculations for DMC, Li^+DMC and CIP ($[TFSI]^-Li^+$DMC).

Table S1 Calculated transition wavelengths, oscillator strength, main initial states, and main final states of DMC.

Wavelength / nm	Oscillator strength	Initial state	Final state
145.4	0.0239	HOMO	LUMO+3
141.8	0.0312	HOMO-1	LUMO+2
140.1	0.1336	HOMO-1	LUMO+8
137.2	0.0988	HOMO-2	LUMO+2
134.1	0.0347	HOMO-2	LUMO+2
133.2	0.0544	HOMO-3	LUMO

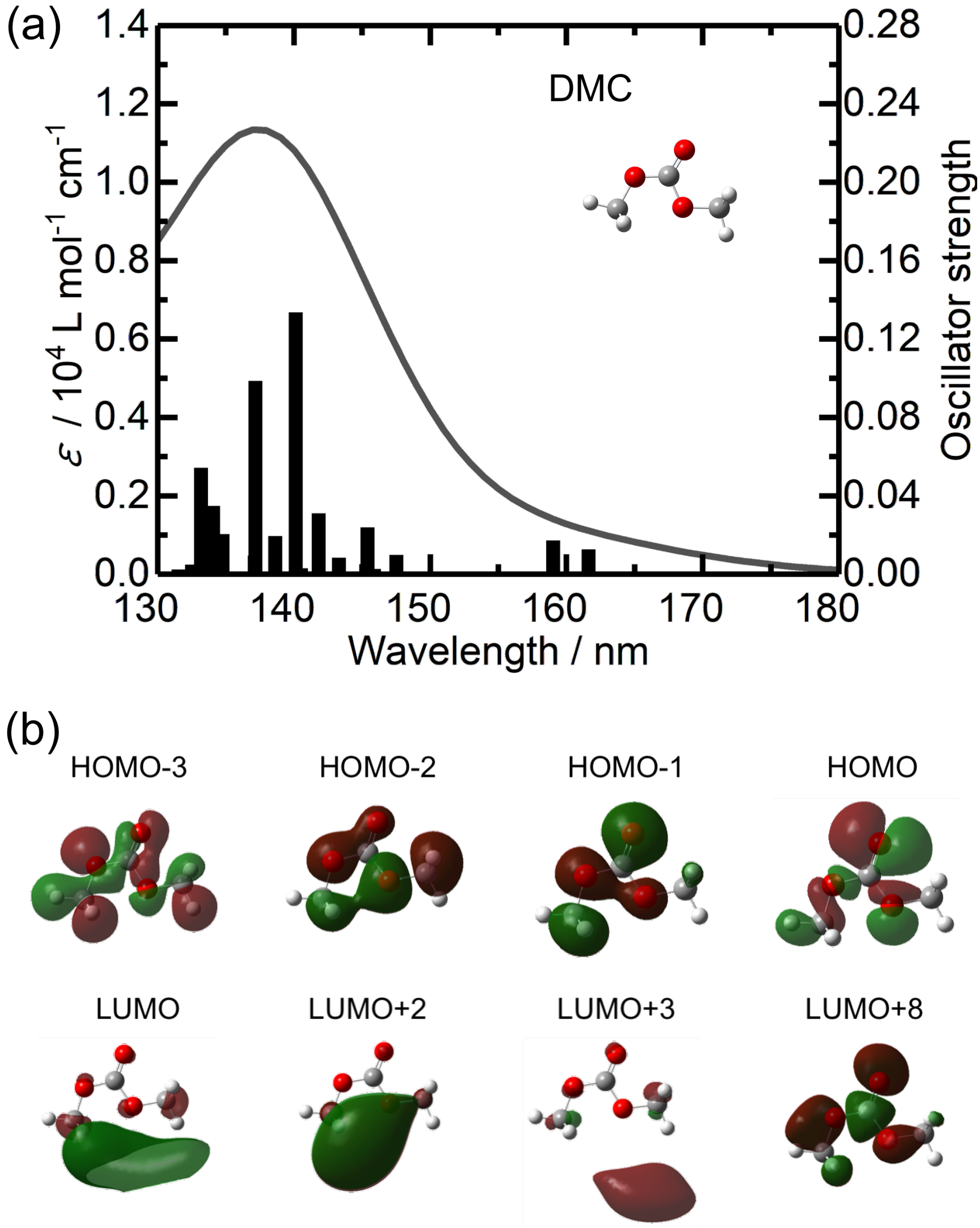

Fig. S7 (a) Calculated oscillator strengths, molar extinction coefficients (ε), and **(b)** visualized molecular orbitals of the orbitals listed in Table S1.

Table S2 Calculated transition wavelengths, oscillator strength, main initial states, and main final states of $Li^{+}DMC$.

Wavelength / nm	Oscillator strength	Initial state	Final state
148.1	0.2331	HOMO-1	LUMO+1
142.8	0.1828	HOMO-1	LUMO+1
132.8	0.0144	HOMO-3	LUMO
130.8	0.0328	HOMO-2	LUMO+2

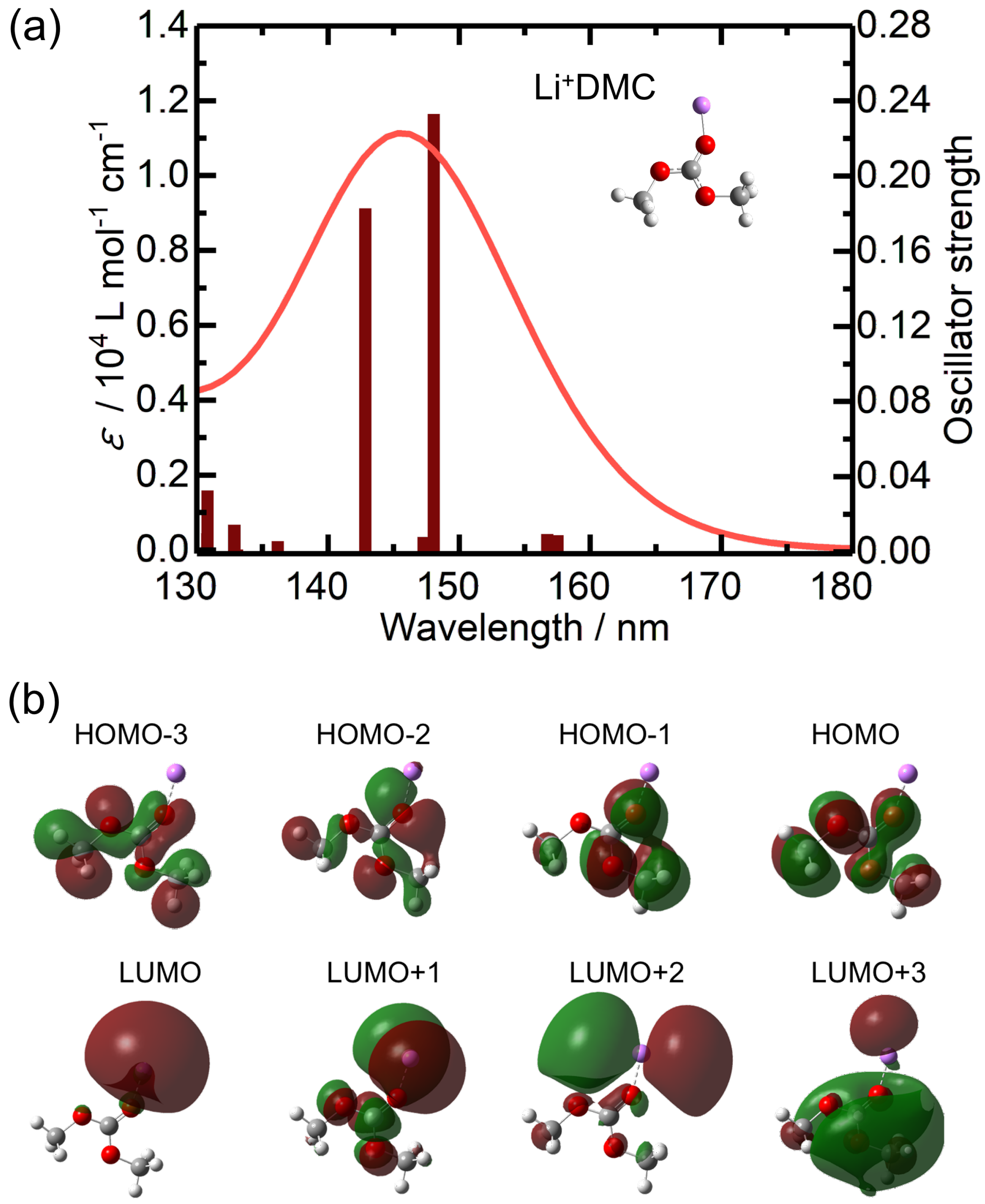

Fig. S8 (a) Calculated oscillator strengths, molar extinction coefficients (ε), and **(b)** visualized molecular orbitals of the orbitals listed in Table S2.

Table S3 Calculated transition wavelengths, oscillator strength, main initial states, and main final states of CIP ($[TFSI]^{-}Li^{+}DMC$).

Wavelength / nm	Oscillator strength	Initial state	Final state
141.0	0.1523	HOMO	LUMO+3
141.9	0.0792	HOMO	LUMO+3
142.2	0.0630	HOMO	LUMO+3
142.8	0.3225	HOMO-6	LUMO+8
143.8	0.0344	HOMO-2	LUMO
145.6	0.0462	HOMO	LUMO+1
147.8	0.0204	HOMO-1	LUMO
158.9	0.0194	HOMO	LUMO+16

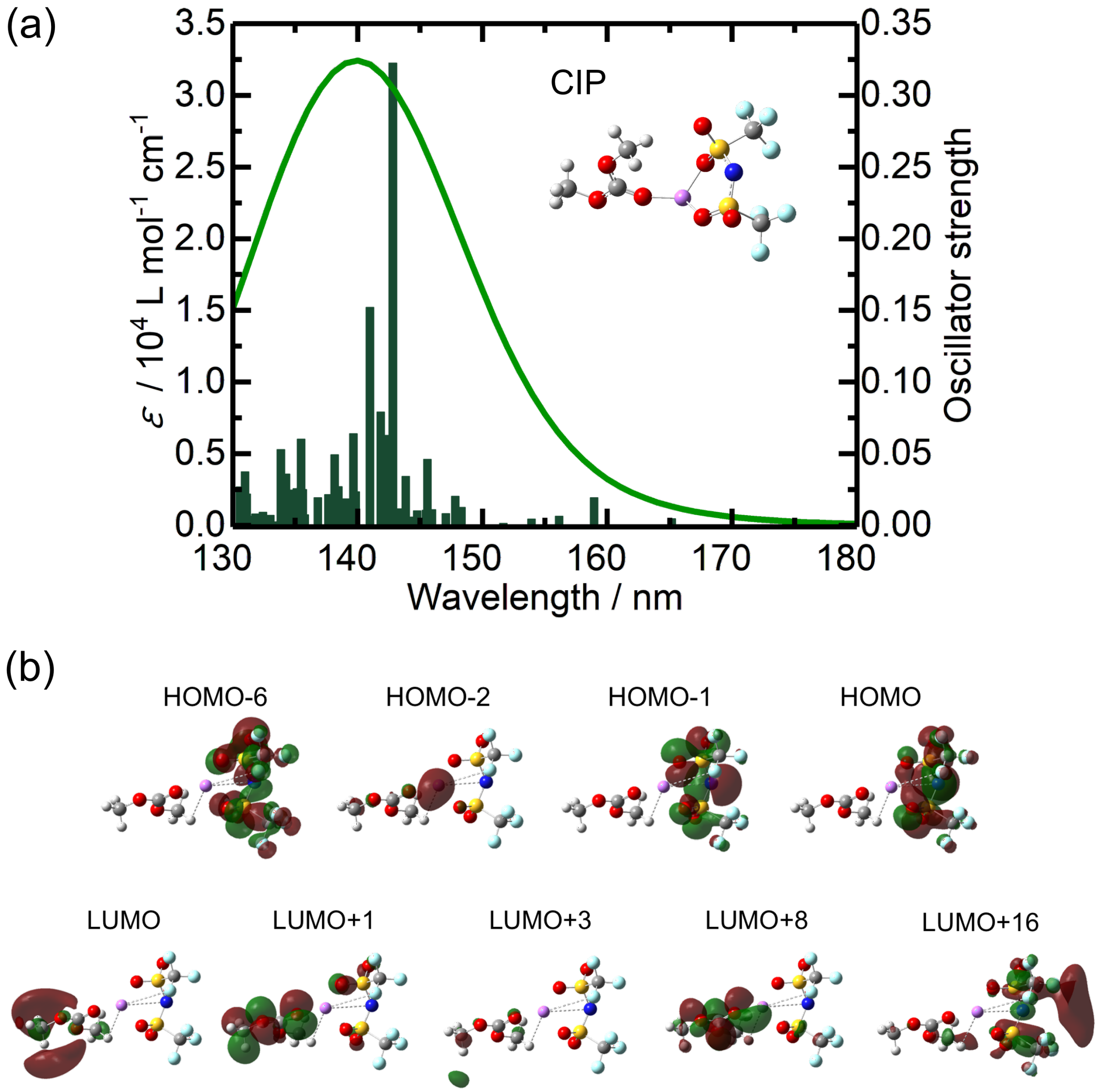

Fig. S9 (a) Calculated oscillator strengths, molar extinction coefficients (ε), and **(b)** visualized molecular orbitals of the orbitals listed in Table S3.

In the previous paper,[S1] by utilizing the TD-DFT calculations, the absorption spectrum in the FUV region of pure DMC was successfully reproduced and assigned to the electronic transition within DMC (Table S1 and Fig. S7), and the charge transfer between DMC and Li^+ in the electrolyte was revealed as shown in Table S2 and Fig. S8. In the low-concentration region, the spectral changes could be explained by the contributions of these two components: free DMC and Li^+DMC. However, in this study, as the Li^+ concentration increased, the influence of the anion on the spectrum also became evident. Consequently, it was revealed that, in addition to the interaction between Li^+ and DMC, absorption arising from electron transfer from the anion to DMC also contributes to the spectrum.

References

S1 H. Sato, N. Ueno and I. Tanabe, *Chem Commun.*, 2024, **60**, 6375.
